# Inhibition of pathogenic bacterial biofilms on PDMS based implants by *L. acidophilus* derived biosurfactant

**DOI:** 10.1186/s12866-019-1412-z

**Published:** 2019-02-13

**Authors:** Surekha K. Satpute, Nishigandha S. Mone, Parijat Das, Ibrahim M. Banat, Arun G. Banpurkar

**Affiliations:** 1 Department of Microbiology, Savitribai Phule Pune University, Pune, Maharashtra 411007 India; 2 Department of Physics, Savitribai Phule Pune University, Pune, Maharashtra 411007 India; 3 School of Biomedical Sciences, University of Ulster, Coleraine, BT52 1SA, N., Ireland, UK; 4 Present Address: Protein Crystallography lab (603), Department of Biosciences and Bioengineering, Indian Institute of Technology Bombay, Powai, Mumbai, Maharashtra 400076 India

**Keywords:** Adhesion, Biosurfactant, *Lactobacillus*, Biofilm, Medical implants, PDMS, protein, surface tension

## Abstract

**Background:**

*Lactobacillus* spp. predominantly shows its presence as a normal mucosal flora of the mouth and intestine. Therefore, the objective of our research is to investigate the in-vitro conditions for the prospective of medically valuable biosurfactants (BSs) derived from *Lactobacillus* spp. Biosurfactant (BS) obtained from *Lactobacillus* spp. exhibit antibiofilm and antiadhesive activity against broad range of microbes. In the present study we investigated the production, purification and properties of key components of the cell-associated-biosurfactant (CABS) from *Lactobacillus acidophilus* NCIM 2903.

**Results:**

Extracted, purified, freeze-dried CABS shows reduction in surface tension (SFT) of phosphate buffer saline (PBS @pH 7.0) from 71 to 26 mN/m and had a critical micelle concentration (CMC) of 23.6 mg/mL. The CABS showed reduction in interfacial tension (IFT) against various hydrocarbons and had effective spreading capability as reflected through the decrease in contact angle (CA) on different surfaces (polydimethylsiloxane - PDMS, Teflon tape, glass surface, polystyrene film and OHP sheet). The anionic nature of CABS displayed stability at different pH and temperatures and formed stable emulsions. Thin layer chromatography (TLC) and Fourier transform infrared spectroscopy (FTIR) revealed CABS as glycolipoprotein type. The Sodium Dodecyl Sulphate Polyacrylamide Gel Electrophoresis (SDS-PAGE) showed presence of multiple bands in a molecular range of 14.4 to 60 kDa, with prominent bands of 45 kDa. The CABS has significant antiadhesion and antibiofilm activity against tested bacterial strains.

**Conclusion:**

The current challenging situation is to develop methods or search for the molecules that will prevent the formations of biofilm on medical bioimplants of PDMS based materials. These findings are supportive for the use of Lactobacilli derived BS as potential antiadhesive agent on various surfaces of biomedical devices.

## Background

Biosurfactants (BSs) are amphiphilic molecules produced by diverse microorganisms such as bacteria, fungi and yeasts at the microbial cell surface or excreted out with pronounced surface and emulsifying activities [1–3]. These molecules possess a tendency to accumulate at the interface between liquid phases that show different degrees of polarity and hydrogen bonding, like oil-water or air-water, and reduce the surface and interfacial tension [4]. Microbial biosurfactant (BS) reveal a wide diversity of chemical structures, viz., glycolipids, lipopeptides, polysaccharide–protein complexes, lipopolysaccharides, fatty acids, phospholipids, and neutral lipids [5], therefore, it is reasonable to expect diverse potential applications for different groups of BS in industrial, environmental, biomedical and therapeutic purposes [4, 6, 7]. BS are utilized in various industrial domains including food and cosmetic industries. Extraordinary properties like (i) emulsification (ii) de-emulsification (iii) dispersion (iv) foam formation (v) moisturizing effects are the center of attractions to many researchers. Due to their other properties like (i) pore formation ability and (ii) destabilization of biological membrane; BS are widely explored as antimicrobial, antiviral, antitumor, hemolytic and insecticidal agents in therapeutics. Additionally, other attractive features like (i) viscosity reduction, (ii) hydrocarbon solubilization and (iii) metal sequestering abilities of BS present them as a potential candidate for environmental applications [8].

The BS synthesis has been reported by various genera of bacteria, viz., *Pseudomonas*, *Bacillus*, *Acinetobacter*, *Arthrobacter*, *Rhodococcus*, *Clostridium*, *Halomonas*, *Lactobacillus*, *Leuconostoc*, *Myroides*, and *Serratia*, also in some genera of yeast viz.*, Debrayomyces and Rhodotorula* [3]. *Lactobacillus* gained importance in biomedical and therapeutic field for the exploration of BS synthesis due to two reasons, (i) they constitute an important part of natural microflora [9] and (ii) they possess antiinfective properties [10]. Whereas the indigeneity of *Lactobacillus* in the microflora of healthy human play a key role in maintaining stability and diversity of the gut microbiome [11], the antiinfective properties of *Lactobacillus* prevent the enteropathogen-mediated infection by competing for nutrients and binding sites (e.g., inducing intestinal mucin gene expression), by secreting antimicrobial substances such as (i) organic acids, (ii) H_2_O_2_, (iii) bacteriocins and reducing gut pH and producing BSs, eventually by counteracting the spread within the colonized body [12]. Unlike BS derived from *Lactobacillus,* BS derived from other microorganisms may raise the issue of health risk. For example, *Serratia marcescens* produce serrawettin which plays some role in the virulence of this species [13]. Likewise, *Pseudomonas* strains producing rhamnolipids exhibit pathogenic potential, suggesting that these biomolecules may contribute to its opportunistic pathogen characteristics [14]. Therefore, synthesis of BS from *Lactobacillus* is a subject of research interest. Different species of *Lactobacillus* viz., *L. plantarum* [15], *L. agilis* [16], *L. paracasei* [17], *L. pentosus* [18], *L. casei* [19], *L. helveticus* [20], *L. fermenti* [21], *L. rhamnosus* [21], *L. acidophilus* [22] have been reported for synthesis of various types of BS, such as glycoprotein, glycolipid, glycolipoprotein. *Lactobacillus* derived BS are increasingly applied as antiadhesive agents in therapeutic field, which play an important role in the prevention and control of infections caused by biofilm forming pathogens from various groups of microbes [23–25]. Biofilms are communities of microorganisms that are encased in a self-synthesized extracellular polymeric matrix, and grow attached to a biotic or abiotic surface [26]. Biofilms often act as reservoirs of pathogenic microorganisms. The biofilm matrix protects the enclosed microorganisms by (i) increased access to nutrients (ii) reduction or delay in the penetration of antimicrobials and toxins (iii) continuance of extracellular enzyme activities and (iv) shelter from predation [26]. Formation of biofilm on or within indwelling medical devices poses a critical problem for medical care. The inherent resistance of biofilms has prompted research in the development of antiadhesive biological agents from BS to disrupt biofilms. Thus, the current research depicts the evidences in preventing the colonization of biofilm on PDMS based implant materials. Therefore, in this study, we explored the BS from *L. acidophilus* NCIM 2903 and structurally characterized for antibiofilm properties using various model organisms.

## Methods

### Strains and standard culture conditions

*L. acidophilus* NCIM 2903 was procured from the National Collection of Industrial Microorganisms (NCIM), National Chemical Laboratory (NCL), Pune, Maharashtra, India. For antibacterial and antiadhesive assays, *Escherichia coli* NCIM 2065, *Staphylococcus aureus* NCIM 2079, *Proteus vulgaris* NCIM 2027 cultures were all obtained from NCIM, NCL, Pune, India. Other cultures viz., *Bacillus subtilis* MTCC 2423, *Pseudomonas putida* MTCC 2467 and *P. aeruginosa* MTCC 2297 were obtained from Microbial Type Culture Collection - MTCC, Chandigarh, India. NCIM 2903 was grown and maintained in the laboratory on DeMan, Rogosa and Sharpe (MRS) (Himedia) agar [27]. The strain NCIM 2903 was previously isolated from curd sample. The standard temperature suggested for growth of this organism is 37 °C on MRS medium. Composition of MRS medium is as follows - Proteose peptone 10.000, Beef extract 10.000, Yeast extract 5.000, Dextrose 20.000, Polysorbate 80 1.000, Ammonium citrate 2.000, Sodium acetate 5.000, Magnesium sulphate 0.100, Manganese sulphate 0.050, Dipotassium phosphate 2.000 (g/L) final pH 6.5 ± 0.2. All other cultures were also grown and maintained in nutrient broth as per supplier’s instructions.

### Assessment of biosurfactant biosynthesis

The BS production was assayed by different screening methods, since no single method is sufficiently accurate to assess the BS biosynthesis. The methods used in the study are as follows: Drop collapse test (DCT), Hemolytic Activity (HA), Emulsification Index, (EI), Oil Spread Method (OSM), Blue Agar Plate method (BAP), Tilted Glass Slide Test (TGST). All methods were carried out as described from our previous work [28]. Apart from these all aforementioned methods, we also assessed the BS biosynthesis by measuring the surface tension (SFT) of by a pendant drop technique using Optical Contact Angle Goniometer (OCA 15+, DataPhysics Instruments GmbH, Germany). In this method, the equilibrium shape of the pendent drop in the gravitational force was captured using charge-coupled device (CCD) camera and analyzed in real time using SCA 20 software. The axisymmetric pendant drop shape analysis was carried out by using Laplace-Young’s equation for determining the SFT values [29, 30].

### Biosurfactant production

For production of BS three different media were used, viz., (i) Fermentation medium (FM) (designed by us -composition g/L-Peptone: 10.000, Beef extract: 10.000, Yeast extract: 10.000, Tri sodium citrate: 5.000 at pH 6.5, (ii) MRS with Tween 80 (MRS-T) (0.1%), (iii) MRS without Tween 80. The media were examined for better production of BS and the examination for the same was done by measuring the SFT by pendant drop method at different time intervals. Further, FM medium was selected for the BS production throughout the research. A seed culture was prepared by transferring a single colony of *L. acidophilus* into 10 ml of fermentation medium (FM) and incubated it overnight at 30 °C under shaking conditions (170 rpm). For BS production, a 5% (*V*/V) seed culture was transferred into sterile 150 ml of FM in 1 L Erlenmeyer flask and incubated at 170 rpm and 30 °C up to 120 h. The BS biosynthesis was monitored after every 12 h. The biomass during the BS biosynthesis was determined as dry cell weight. For this purpose, a volume of 10 ml of FM was filtered through sterile filter paper (0.22 μm Millipore, Bangalore, India) and dried at 105 °C for 24 h and reweighed. Dry bacterial biomass was repeatedly weighed till constant weight was achieved.

### Extraction of biosurfactant

For the extraction of cell CABS, cells were harvested by centrifugation (15,000 *g*, 20 min, 4 °C), washed in demineralized water and resuspended in 50 mL of phosphate buffered saline (PBS, 0.01 M KH_2_PO_4_/K_2_HPO_4_ and 0.15 M NaCl with pH adjusted to 7.0). Cells were gently resuspended at room temperature (RT) of 30 °C for 2 h to release BS associated with bacterial cell followed by centrifugation (15,000 *g*, 20 min, 4 °C). Remaining supernatant was filtered through filter (0.22 mm pore size, Millipore, Bangalore, India). The supernatant was dialyzed against demineralized water with dialysis membranes having molecular mass cut-off 12,000 Da (Sigma-Aldrich, USA) and further freeze-dried and stored at − 20 °C until further use.

### Physical characterization of biosurfactant

It was determined by measuring the physical properties of the BS viz., ionic character (IC), relative emulsion volume (REV), temperature and pH stability. Importantly, other physical properties, SFT, critical micelle concentration (CMC) and also interfacial tension (IFT) was determined by a pendant drop technique using OCA Goniometer. The spreading capacity of BS was determined by measuring the contact angle (CA) of CMC solution of BS in a sessile drop technique. Synthetic surfactants (their CMC concentrations) viz., cetyl trimethyl ammonium bromide (CTAB), Aerosol-OT/sodium bis (2-ethylhexyl) sulfosuccinate (AOT), Sodium dodecyl sulphate (SDS), Tween 80 were included as reference surfactants for testing all physical properties of BS.

### Critical micelle concentration (CMC) of biosurfactant

The CMC value of BS was determined by measuring the SFT of BS in PBS solution (pH 7.0). The CMC is the point where an increase in the concentration of BS does not have any further reduction in SFT. In this technique, drop of liquid of different concentrations of BS, ranging from 1 to 60 mg/ml prepared in PBS was allowed to hang from the end of a capillary and the asymmetric shape of a pendant drop was determined by using Laplace-Young equation [29, 30] for measuring the value of SFT. The measurements of CA were made by using OCA Goniometer. The CMC was determined by plotting the SFT as a function of the logarithm of BS concentration and is represented as the point at which the baseline of minimal SFT intersects the slope where SFT shows a linear decline.

### Determination of hydrocarbon-water interfacial tension (IFT)

The IFT of the BS sample was also determined. A glass cuvette (*l* = 35 mm, *b* = 22 mm and, *h* = 25 mm) was cleaned in ultrasonic bath using aqueous detergent solution, followed by washing with distilled water, acetone, ethanol and finally isopropyl alcohol. After cleaning and drying procedures, the glass cubical was filled with 15 ml of kerosene (purchased from local market) and placed on the platform of Goniometer. Further, a syringe was filled with reference and BS solutions (at CMC concentration) and the tip of the syringe was dipped completely in kerosene filled in the glass cubical. Subsequently a pendent drop of BS solution in kerosene was formed, and measurements for IFT were taken between two immiscible liquids as discussed earlier.

### Determination of spreading capacity of biosurfactant

The spreading ability or wettability of BS was determined by measuring the contact angle (CA) on various surfaces by using sessile drop techniques A drop of 25 μl of sample was placed and measurements were taken on five different surfaces viz., Teflon polymer (Polytetrafluoroethelene-PTFE) tape (highly hydrophobic), Polydimethyl siloxane (PDMS) (highly hydrophobic), polystyrene, overhead projector (OHP) transparency sheet (Intermediate hydrophilic-hydrophobic) and glass slide (highly hydrophilic) surfaces.

### Analysis in change of the hydrophobicity of PDMS surface through coating of biosurfactant

The CA measurements were taken on both PDMS surface (Control) and PDMS coated with BS (Test). A thin layer CABS (CMC solution 23.6 mg/ml) was coated on PDMS surface by immersing the PDMS sections (*l* = 2.0 cm, *b* = 2.0 cm, *h* = 0.1 cm) for overnight and dried then further in dust free airflow. After complete drying procedures, the CA of a drop of 25 μl water, PBS (@pH 7) and various synthetic surfactants (as mentioned earlier) were also measured to analyse any change in the hydrophobicity of PDMS surface before and after coating of CABS.

### Determination of temperature and pH stability

The stability of BS was determined at different pH and temperatures by measuring SFT values. Buffers in the range of 2.0, 4.0 …12.0) were used to examine the stability of BS. Similarly, to evaluate stability at different temperatures, the freeze-dried CABS was dissolved in PBS (pH 7.0) and incubated at 28 °C, 37 °C and 60 °C for 48 h and SFT for each sample was determined before and after incubation procedures. In addition, the activity of BS was also checked before and after autoclaving.

### Relative emulsion volume of biosurfactant and its stability

The emulsion-stabilizing capacity of the BS was tested on five different hydrocarbons (kerosene, n-decane, n-hexane, xylene, benzene and n-heptane). In this technique, 2 ml of BS (CMC solution) and 2 ml each of hydrocarbons were taken in a graduating test tube, shaken vigorously for 2 min, and left stand still for 24 h. The relative emulsion volume (EV in %) and emulsion stability (ES in %) were calculated as per the method described by Das et al. [31].

### Thin layer chromatography (TLC)

Briefly, 10 μl aliquot of BS sample was concentrated on a pre-coated silica gel plate –TLC (Merck, KGaA, Darmstadt, Germany), and run in following system: chloroform: methanol: acetic acid (65: 25: 2); butanol: ethanol: water (5: 3: 2); chloroform: methanol: water (65: 15: 2); butanol: acetic acid: water (12: 3: 5) and developed respectively with anisaldehyde, diphenylamine, iodine and ninhydrin reagents for the respective determination of glycolipid, sugar, lipids and amino acid moieties of BS.

### Fourier trans-form infrared spectroscopy (FT-IR) of functional groups

The FTIR facilitates identification of types of chemical bonds i.e. functional groups present in BS. One milligram of BS powder was grounded with 100 mg of potassium bromide (KBr) and translucent pellet was analyzed using FTIR device (Jasco FT/IR-6100, Japan). The spectrum in the range of 400–4000 cm^− 1^ at a resolution of 4 cm^− 1^ was obtained and analysed.

### Determination of molecular weight of protein by sodium dodecyl sulphate polyacrylamine gel electrophoresis (SDS-PAGE)

Gel electrophoresis of BS (50 μg) was carried out using 12% (*w*/*v*) resolving gel and 4% stacking gel and run at a constant current (25 mA) till the dye front reaches the bottom. The gels were developed by staining with Coomassie blue R250. The molecular weight of protein was determined by comparing it with the protein ladder. The protein ladder (Bio-Rad, US) used in the study is as follows: (weights in kDa) β-Galactosidase (116.2); Phosphocyclase –B (97.4); Serum albumin (66.2); Ovalbovine (45); Carbonic unhydrase (31); Trypsin inhibitor (21.5); Lysozyme (14.4).

### Determination of ionic character

Agar double diffusion technique was used to determine the ionic charge of BS [32]. For this study, two regularly-spaced rows of wells were made in an agar of low hardness (1% agar *w*/*v*). The lower row of wells was filled with the BS solution (at CMC concentration) and the upper rows of well were filled with reference surfactants (SDS: 20 mM and AOT: 20 mM, CTAB: 20 mM and Barium chloride: 20 mM and Triton X – 100: 0.9 mM and Tween 80: 0.012 mM each) of different ionic charges, and monitored for the presence of precipitation line over a period of 48 h at RT.

### Antibacterial and antiadhesion assay

Antibacterial and antiadhesion assays of BS were performed by using six bacterial cultures viz., *E. coli*, *S. aureus*, *P. vulgaris*, *B. subtilis*, *P. putida*, and *P. aeruginosa*. Micro-dilution technique in 96-well flat-bottom plastic tissue culture plates (Tarsons, India) was carried out to determine the antibacterial and antiadhesive activity of BS against selected bacterial cultures [17].

### Preparation of polydimethylsiloxane (PDMS) based contact lens surface

PDMS based contact lenses were prepared by mixing 184 silicone elastomeric base with a curing agent (10:1) (Elastomer solution kit: 184 Sylgard, Dow Corning, Rheingaustrasse, Wiesbaden, Germany) followed by gentle stirring to obtain a uniform and clear solution. The clear solution of PDMS was poured in sterile disposable petri dish. It was allowed to solidify at temperature of 80–100 °C for 6 h in vacuum oven at a pressure of 100 Pa (10^**− 3**^ to 1 Torr). Circular PDMS discs (diameter 0.8 cm and thickness of 0.1 cm) were cut and used for evaluating adhesion inhibition activity of BS against pathogenic bacteria.

### Inhibition of bacterial pathogen adhesion on PDMS based surfaces treated with biosurfactant

Two sets of sterile PDMS discs were used to demonstrate the antiadhesion and antibiofilm effect of formulation. One set without any BS coating was considered as control. The other set coated with BS (CMC solution in buffer) was considered as test. For coating, all discs were immersed in sterile nutrient culture medium and then inoculated with test culture and incubated at 37 °C. Further discs were removed and rinsed with sterile distilled water to remove un-adhered microorganisms. Further whole discs were observed under light microscope (10 X), and scanning electron microscopic (SEM - JOEL, JSM-6360A) images were also taken.

### Statistical analysis

All experiments were performed in triplicates. The standard deviation of the data was analyzed using the software ‘GraphPad Prism version 7.00 (GraphPad Software, San Diego, CA)’ and is presented in the form of Figs. *P* values < 0.05 were considered significant. Error bars are represented as as ± SD.

## Results

### Screening for biosurfactant synthesis

The results of the screening assays used to confirm BS production including SFT, CA measurements were positive.

### Medium for biosurfactant synthesis

**Fig. 1:**
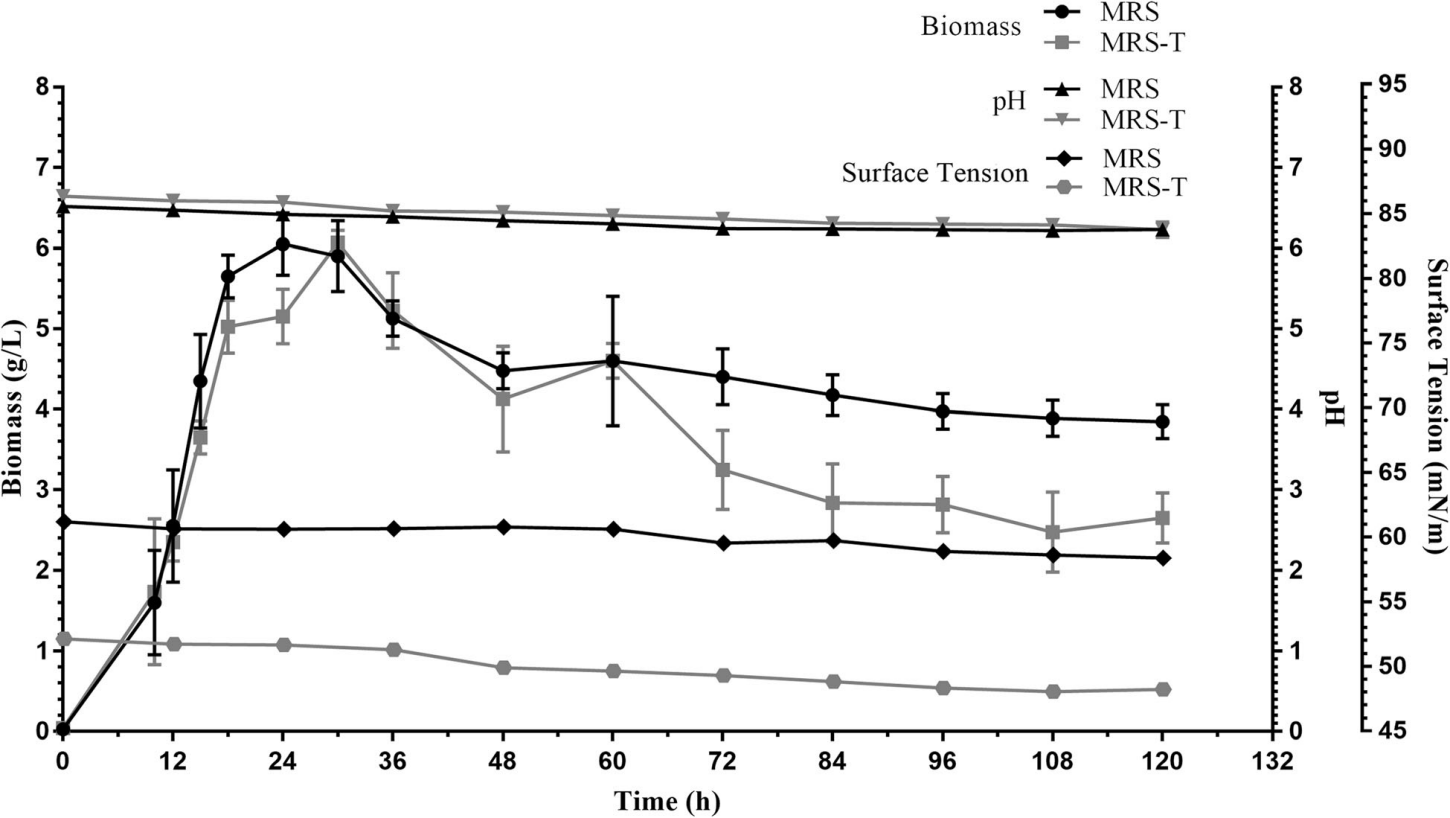
Analysis of surface tension, biomass and pH profile of *L. acidophilus* NCIM 2903 grown in MRS, MRS-Tween

**Fig. 2:**
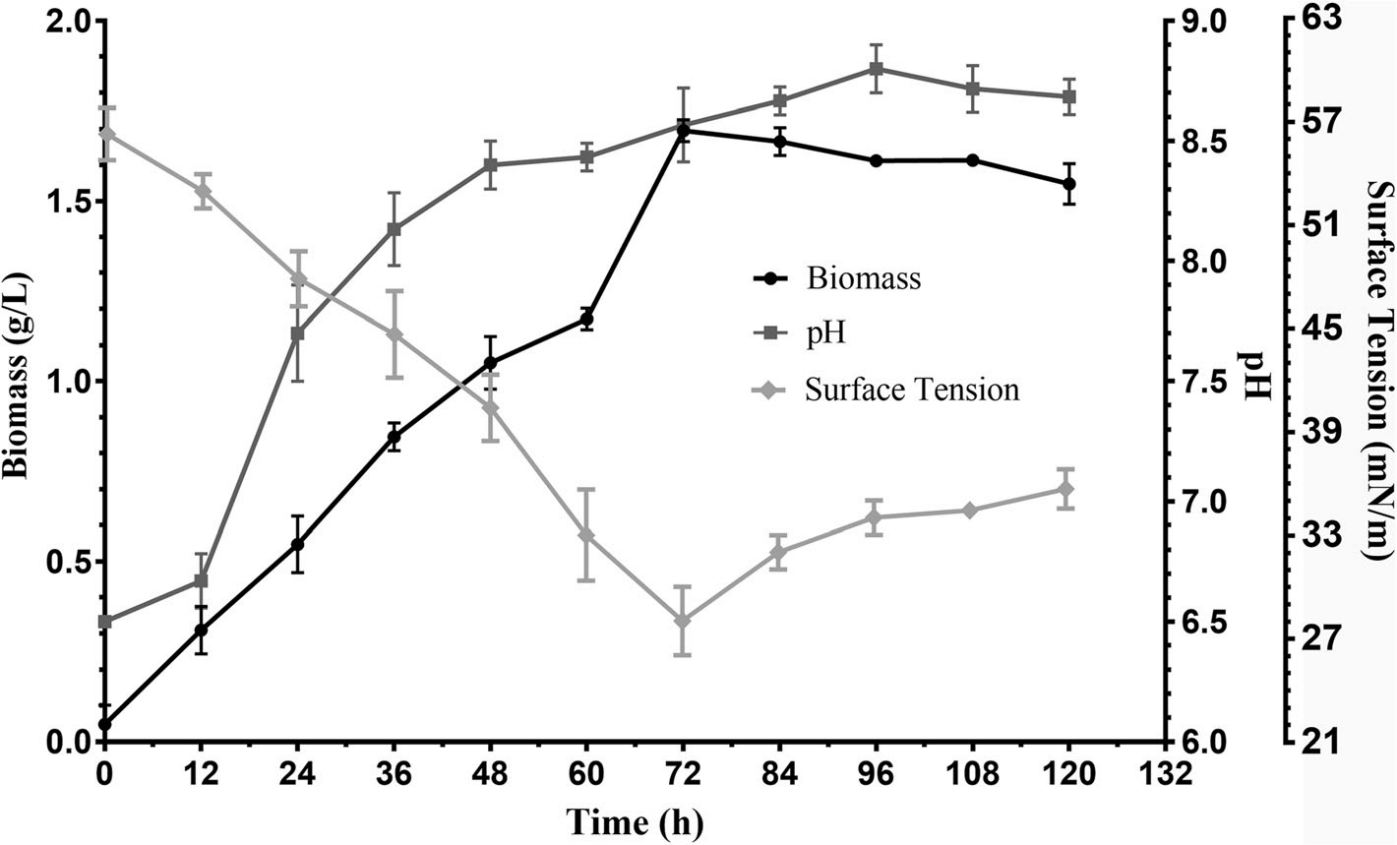
Analysis of surface tension and biomass of *L. acidophilus* NCIM 2903 grown in fermentation medium for production of biosurfactant

BS produced by *L. acidophilus* NCIM 2903 was determined by measuring the SFT of cell free broth and in case of cell bound BS extracted in PBS. In this paper, our work is solely focused on CABS. *L. acidophilus* did not synthesized BS in MRS and MRS-T evident from SFT values. Both MRS and MRS-T supported the growth of *L. acidophilus* culture; however, no drastic change in pH was observed (Refer Fig. 1). Instead it synthesized BS only in FM with an amount of 175 mg/L. The gradual decrease (55 to 28 mN/m) in SFT of FM was observed as the culture continued to grow up to 72 h and further incubation up to 120 h resulted in increase in SFT values (28 to 36 mN/m). The growth of culture (log to stationary phase) was associated with the change in pH (6.5 to 8.7) (Fig. 2). Therefore, all subsequent studies were performed in FM medium.

### Isolation of biosurfactant

The present study is pertinent to BS associated with cells (CABS). The synthesis of BS (evident from SFT values) occurred in FM. At 72 h, the value of SFT reduced from an initial 55 to 28 mN/m. However, after 72 h, the synthesis of BS was decreased (Fig. 2) and was evident from the increased values of SFT 28 to 36 mN/m. During the incubation time, the biomass showed a similar trend, it increased until 72 h and then gradually decreased. The pH of the FM during this period also increased from 6.5 to 8.8.

### Physical properties

Analysis of physical properties of freeze-dried CABS proved it as an effective BS.

### Evaluation of surface tension and critical micelle concentration **(**CMC**)**

**Fig. 3:**
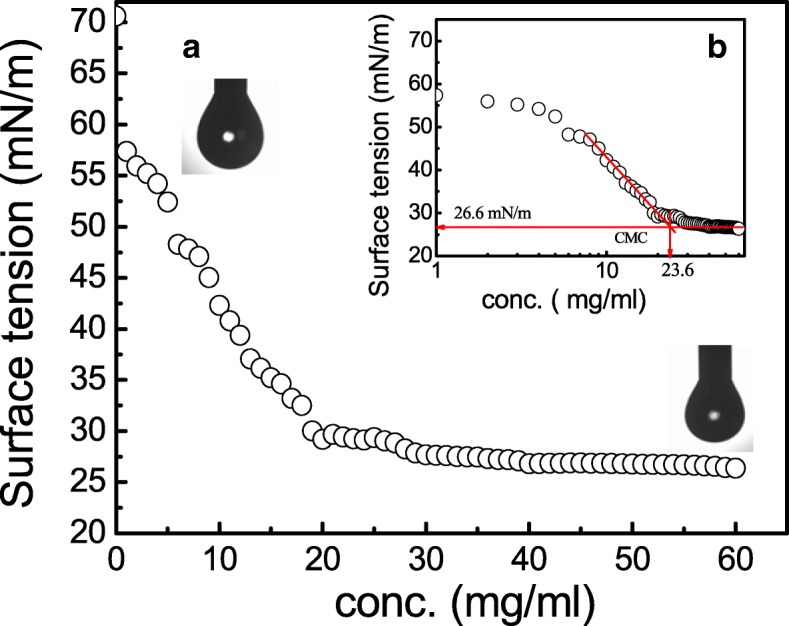
**a** Variation in surface tension and **b** semi-logarithmic reflection of critical micelle concentration (CMC) value from SFT (mN/m) versus BS concentration for CABS extracted from *L. acidophilus* NCIM 2903

The CMC was found out to be 23.6 mg/mL, indicating the concentration of BS after which there is no further reduction in SFT values. CABS could reduce the SFT of the PBS solution from 71 to 26 mN/m (Refer Fig. 3). In addition, the IFT measurements of BS against different hydrocarbons also proved its surfactant property.

### Hydrocarbon-water interfacial tension (IFT) and spreading capacity- contact angle (CA)

**Fig. 4:**
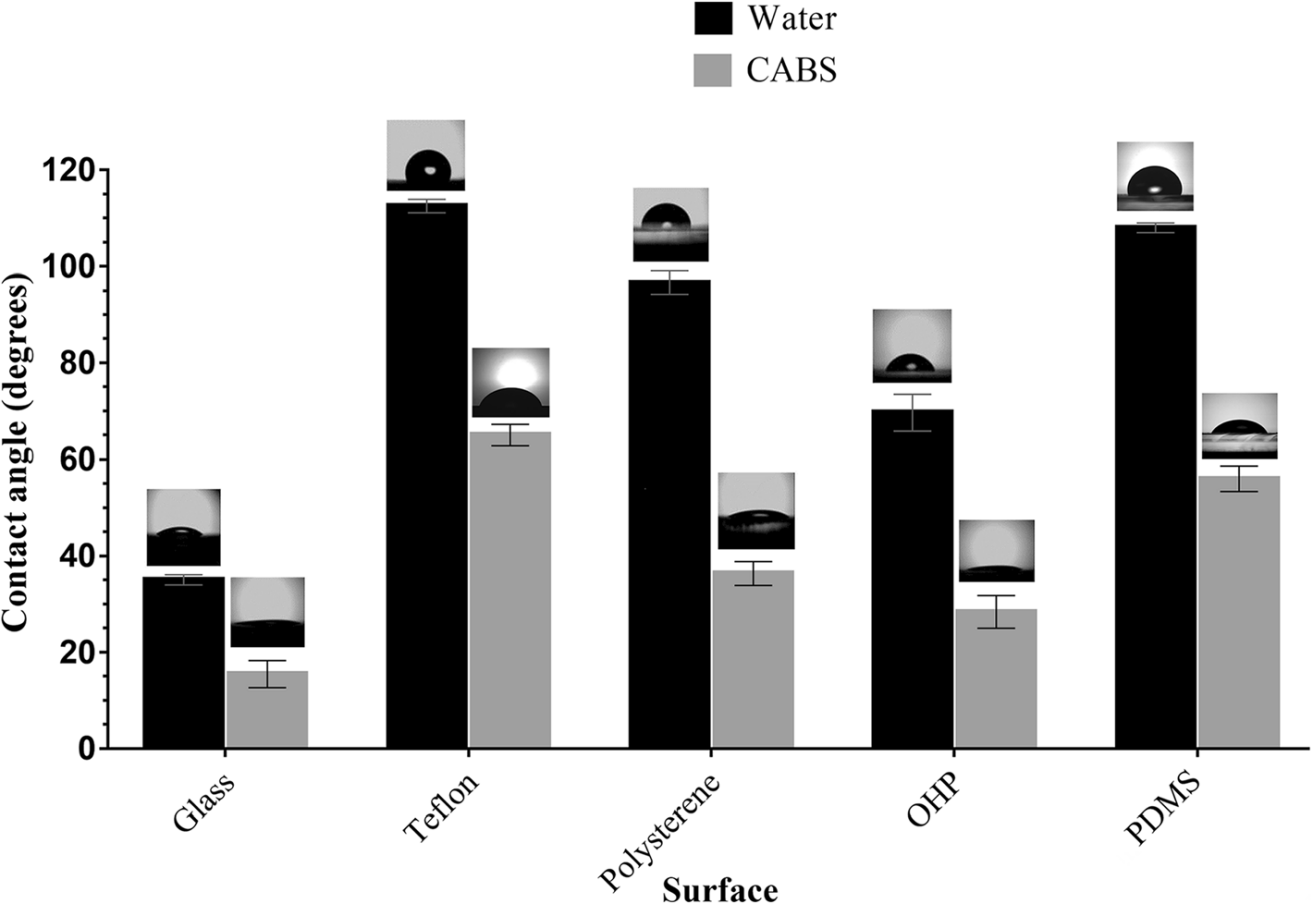
Measurement of Contact angle (CA) for CABS obtained from *L. acidophilus* NCIM 2903 on various surfaces

**Fig. 5:**
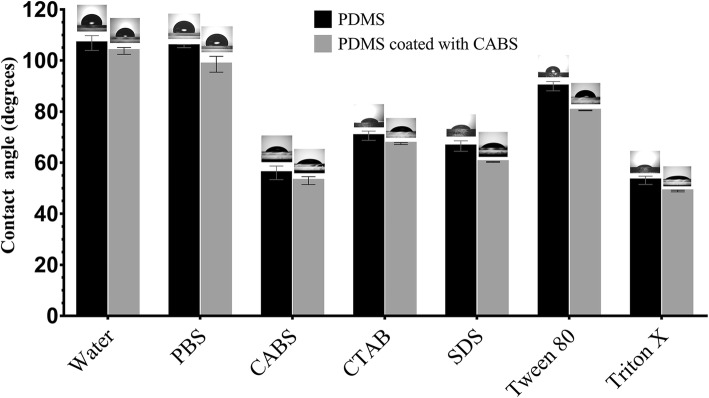
Measurement of Contact angle (CA) on PDMS surface with and without CABS coating (CMC solution 23.6 mg/ml). Data was analysed by paired Student’s t-test

BS showed a reduction in the IFT values with different immiscible liquids where highest reduction being with kerosene interphase, from 27 to 1.5 mN/m. Similarly, the CA measurements of BS on different surfaces are as shown in Fig. 4. The highest reduction in CA was observed on polystyrene surface (from 96 to 36 °) followed by Teflon (from 112 to 65°). On other hydrophobic surface like PDMS the CA was decreased (from 111 to 59°). A marginal reduction in CA (from 35 to 26°) was observed on glass (highly hydrophilic) surface. The results of CA measurements confirmed the good spreading ability of BS which was evident from Fig. 4. When PDMS surfaces were coated with CABS, there was a slight reduction in CA of all samples (*P* < 0.05) (Refer Fig. 5).

### Temperature and pH stability

The values of SFT were stable (26–27 mN/m) and have not changed over a broad range of pH and temperature. The SFT remained relatively stable over a pH range from 6 to 10, and temperature range of 4 °C to 60 °C for 120 h. The SFT values at pH and temperature outside these range increased slightly (31 to 35 mN/m).

### Relative emulsion capacity and its stability

The EC and ES activity of BS against different water immiscible hydrocarbons revealed a relative EV between 25 and 65% and a relative ES between 45 and 87%. The highest EV (65%) and ES (87%) were detected against n-decane, followed by xylene, (46%) and (87%).

### Thin layer chromatography

**Fig. 6:**
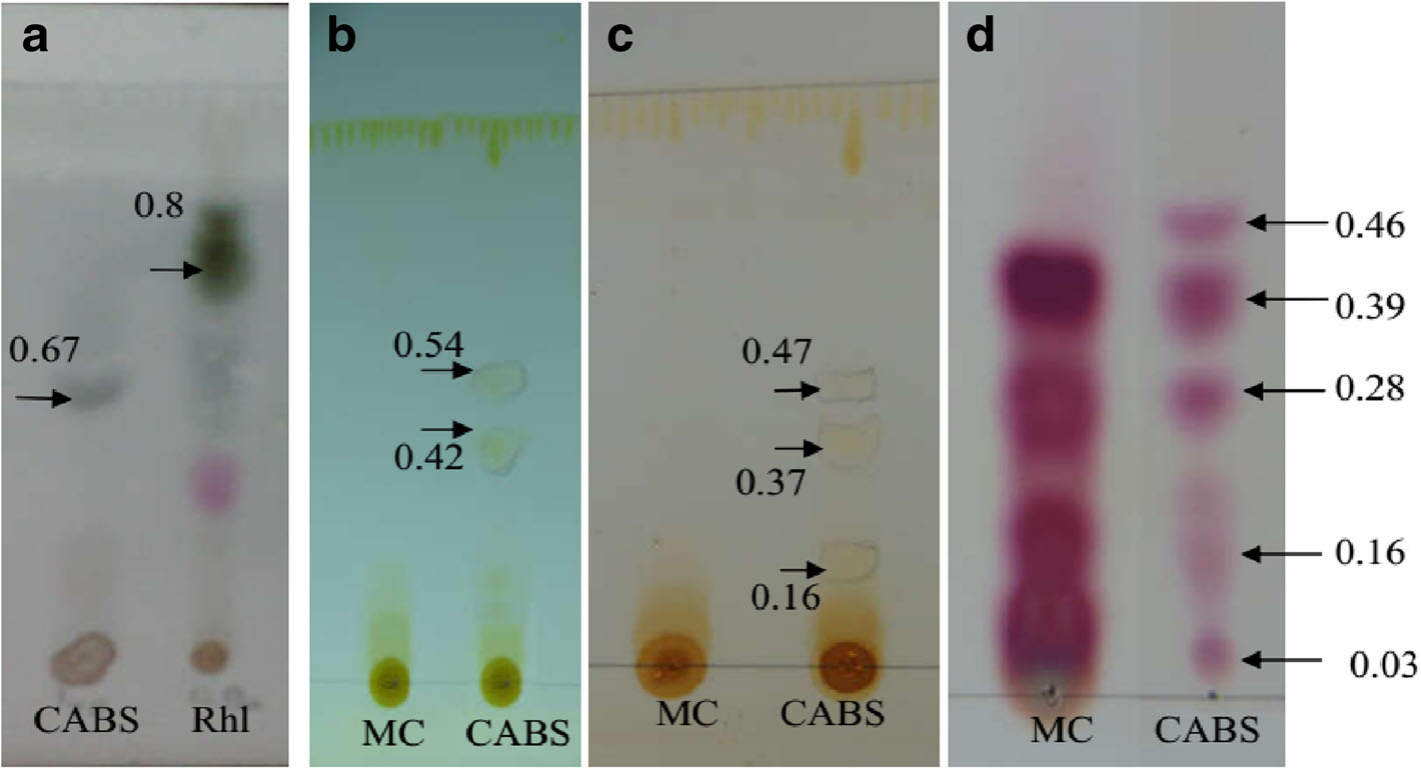
**a**: Sugar; **b**, **c**: Lipid; d: amino acids of CABS obtained from *L. acidophilus* NCIM 2903 which is run in different solvent systems and detected with respective post-chromogenic developing agents. Arrows indicates the presence of various components of CABS detected on TLC sheets. CABS: cell associated biosurfactant; MC: medium control; RHL: Rhamnolipid

The comparison of the BS with reference compounds on TLC showed the presence of sugar (Fig. 6a), lipid (Fig. 6b and c) and amino acids (Fig. 6d). The presence single spot with Rf value of 0.67 confirmed the presence of sugar. Two difference solvent systems used for detection of lipids also indicated presence of yellow brown spots (Rf value 0.54, 0.42 and 0.37, 0.47, 0.16). TLC used for detection of amino acids indicated presence of five pink coloured spots.

### Fourier trans-form infrared spectroscopy (FT-IR)

**Fig. 7:**
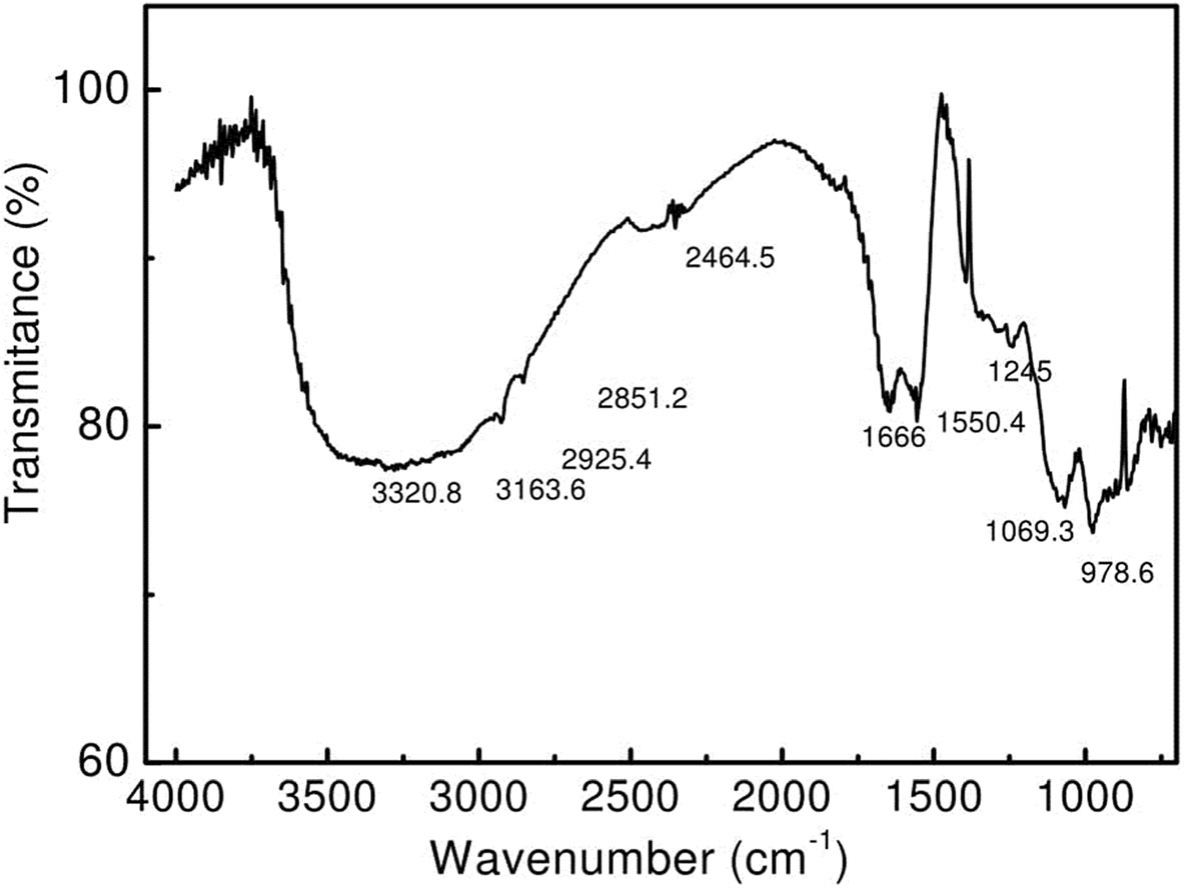
FTIR spectrum of the freeze-dried CABS obtained from *L. acidophilus* NCIM 2903

The molecular composition of BS was further studied by FTIR spectroscopy (Fig. 7). The FTIR shows the stretch between 3400 to 2400 cm^− 1^ confirming the presence of –OH groups while the small peak at 2851 cm^− 1^ corresponds to the existence of C-H bond. Peak at 1666 cm^− 1^ determines C=O stretching while peak at 1550 cm^− 1^ determines presence of -NH stretching, both confirming the presence of proteins. The peak at 1069 indicates the presence of C–O stretching in sugars. Peak at 978 cm^− 1^ reveals the presence of mono-substituted alkenes. The FTIR spectrum obtained in present study suggests the glycolipoprotein type of BS.

### Sodium dodecyl sulphate polyacrylamine gel electrophoresis (SDS-PAGE)

The SDS-PAGE of the BS illustrated multiple bands in a molecular range of 14.4 to 60 kDa, with prominent bands of 45 kDa. It might be because of contamination of peptides which could not be removed completely during the purification process. Please note that the BS reported in this study is cell associated (proteinaceous) and not cell free (not released in the medium), therefore higher is the possibility of cell associated BS got contaminated with other proteins.

### Ionic character

**Fig. 8:**
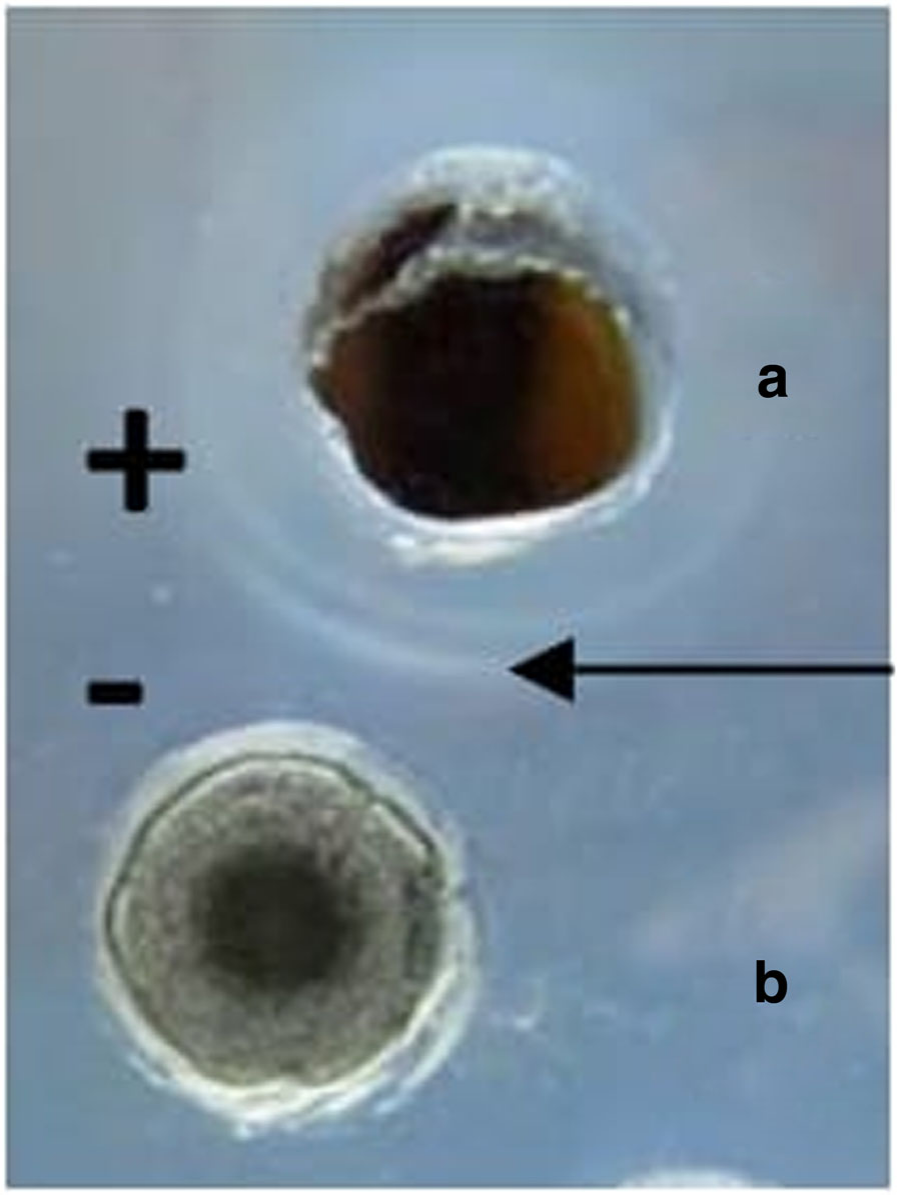
Double diffusion on agar of **a** cationic (positively charged) cetyl trimethyl ammonium bromide (CTAB) against **b** an anionic CABS produced by *L. acidophilus* NCIM 2903; Arrow indicates line of precipitation formed between cationic and anionic pair

Ionic characteristics as revealed by agar double diffusion tests (which are based on the passive diffusion of two compounds possessing charges of the similar or opposite types in a weakly-concentrated gel) displayed precipitation lines between the BS and cationic compounds (CTAB); such precipitation line was not observed between the BS and anionic compounds (SDS) (Fig. 8).

### Antibacterial and antiadhesion activity

**Fig. 9:**
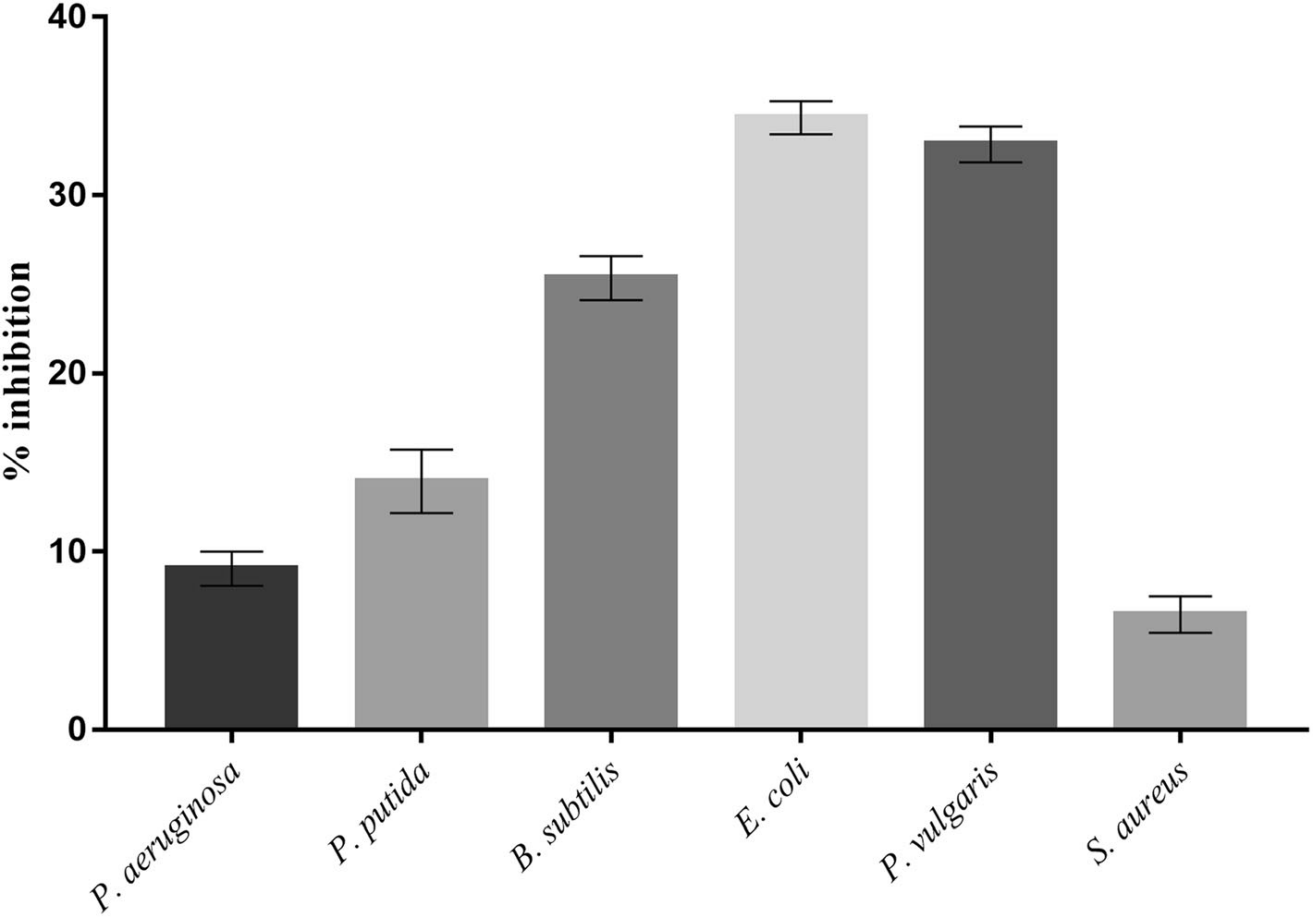
Antibacterial activity of CABS (25 mg/ml) against bacterial pathogens

**Fig. 10:**
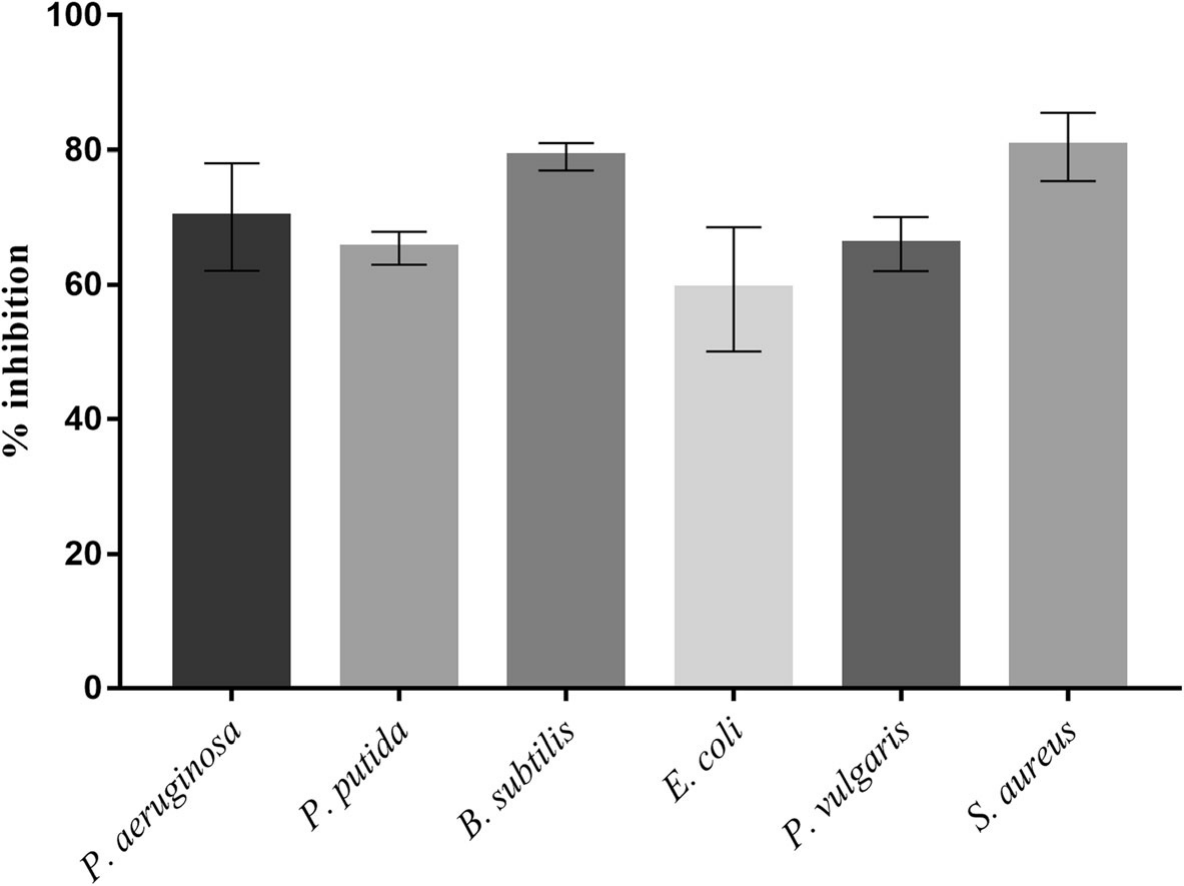
Antiadhesion activity of CABS (25 mg/ml) against bacterial pathogens on polystyrene surface

The BS demonstrated antibacterial and antiadhesive properties. BS at a concentration of 25 mg/mL inhibited the growth of *E. coli* and *P. vulgaris* by 34 and 33% respectively, followed by *B. subtilis* (26%) and *P. putida* (14%) (Fig. 9). Similarly, the BS displayed highest adhesion inhibition of 81 and 79% against *S. aureus* and *B. subtilis* respectively. The adhesion inhibition for remaining four bacterial cultures was found to be in the range of 59 to 65% respectively (Fig. 10).

### Antibiofilm and antiadhesion of biosurfactant on PDMS discs

**Fig. 11:**
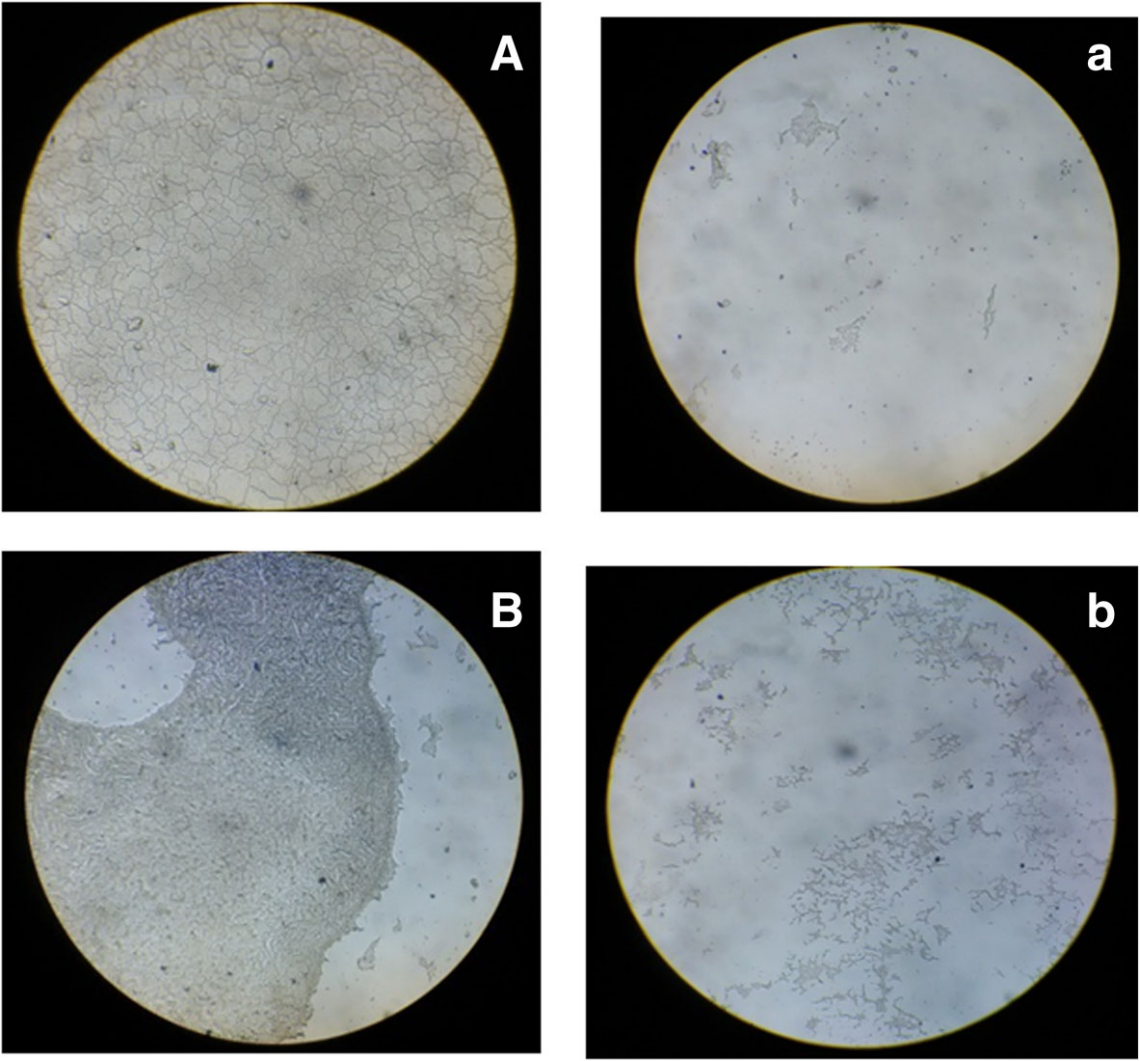
Microscopic images (10X) of biofilm forming bacteria *P. vulgaris* (Control: A) and *S. aureus* (Control: B) on polydimethylsiloxane (PDMS) surface. Impeding effect of *L. acidophilus* NCIM 2903 derived CABS (CMC solution 23.6 mg/ml) on bacterial biofilms of *P. vulgaris* (Test: a) and *S. aureus* (Test: b)

**Fig. 12:**
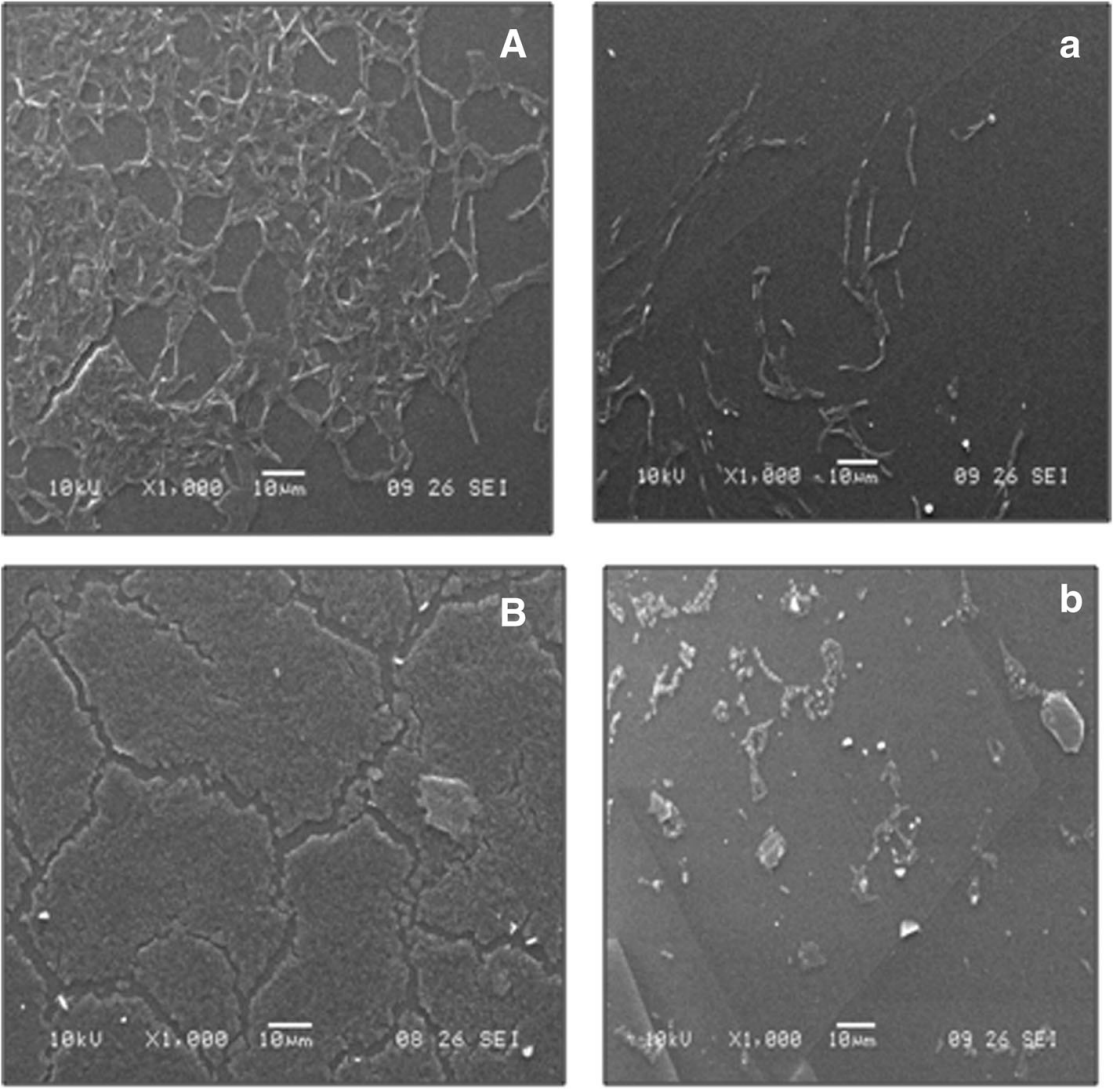
SEM images of biofilm formed by *B. subtilis* (Control: A) and *P. vulgaris* (Control: B) on polydimethylsiloxane (PDMS) based medical implant surface. Impeding effect of *L. acidophilus* NCIM 2903 derived CABS (CMC solution 23.6 mg/ml) on *B. subtilis* (Test: a) and *P. vulgaris* (Test: b) biofilms

Microscopic images (Refer Fig. 11) of biofilm forming bacteria *P. vulgaris* and *S. aureus* on PDMS based contact lenses surface illustrates the antiadhesion property of CABS. SEM studies also demonstrated the antibiofilm and antiadhesive potentials of BS against *P. vulgaris* and *B. subtilis* on PDMS discs (Fig. 12). A massive biofilm was visualized on the PDMS surface without any BS coating, whereas in the presence of the BS coating, a sparse/negligible biofilms were observed. This observation illustrated the adhesion inhibition and antibiofilm potential of the BS.

## Discussion

The ever increasing resistance by pathogenic microbes to current therapeutics agents has drawn attention of researchers towards BSs as the new source of therapeutic agents to control and eradicate such pathogenic microorganisms. BSs have qualified as therapeutics agents due to their ability to (i) disrupt membranes leading to cell lysis and metabolite leakage (ii) disrupt protein conformations of important membrane functions such as transport and energy generation [33] (iii) form a film that changes wettability and surface energy of the original surface affecting the adhesion properties of micro-organisms [34]. Therefore, in present study we explored antibacterial, antibiofilm and antiadhesion properties of the BSs derived from *L. acidophilus* strain. Servin et al. [11] reviewed antagonistic properties of Lactobacilli against microbial pathogens in the gastrointestinal tract. Sharma and Saharan [24] showed antiadhesive and antibacterial potential of BS obtained from *L. helveticus* MRTL91 that was isolated from Chhurpi cheese, against various pathogens of gastrointestinal tract such as *E. coli, S. typhi. Lactobacillus* spp. possesses high capability to inhibit the initial adhesion of uropathogens.

Most of the studies have reported the use of MRS medium for the synthesis of BSs from Lactobacilli spp. [35, 36]. However, in the present study, MRS or its modified media didn’t support the synthesis of BSs in *Lactobacillus* spp. Therefore, we designed a new medium (FM) to support the synthesis of BSs. The decrease in SFT values of FM medium inoculated with *Lactobacillus* culture (test) was compared with control medium (without inoculation). Interestingly, the synthesis of BSs was accompanied by change in the pH of the FM medium from neutral to alkaline condition, which is rather contrary with available reports [20, 37] where respective researchers used deproteinized whey (anaerobic conditions) and MRS-Lac medium (aerobic condition). The production medium used by Sharma et al. [20] contained deproteinized whey and yeast extract (10 g/L) with controlled pH at 6.2 and flushed the BS production medium with N_2_ gas to replace dissolved oxygen. Gudiña et al. [37] used MRS-Lac medium with pH adjusted to 6.2 with incubation of bacterium under aerobic conditions. Both reports [20, 37] have shown a decrease in the pH (neutral to acidic) of the fermentation media during the fermentation process of BSs by *Lactobacillus* genus which might be due to production of lactic acid and other metabolite. However, in present study, an increase in the pH (neutral to alkaline) (Fig. 2) of the FM medium maybe due to accumulations of alkaline byproducts in the culture medium. We have used proteinaceous substrates (yeast extract, beef extract, peptone) which contains several amino acids, peptides, carbohydrates and water soluble vitamins. Proteolysis of such compounds results in the formation of alkaline N-compounds which leads to an overall increase in the pH (alkaline) of FM. Our observations on the onset and maximal level of BSs were contrary to available reports. Reports on the synthesis of BSs suggest the onset and maximal level of BSs in late logarithmic to stationary phases of growth [1, 15, 20, 38]. In our studies we found the synthesis of BSs was growth dependent, and reached maximal at logarithmic growth phase, as witnessed by an increase in the biomass of *Lactobacillus* spp. and change in SFT values. This could have happened due the medium used for the growth of *Lactobacillus* spp. It is likely that FM medium induced the BSs production in the initial logarithmic phase of growth, and continued an increased synthesis of BSs as growth progressed from mid to late logarithmic phase. The change in media composition does affect the production of BS [36, 37]. In fact, the BS production by microorganism is growth associated [37].

It is often important to know the molecular composition of BSs to explore their potential applications in various fields. Therefore, the molecular compositions of the BSs synthesized by *Lactobacillus* were undertaken by performing TLC followed by post chromatographic detection by staining with different chromogenic compounds. The chromatograms of the BSs resemble with the TLC pattern of BSs used as reference, and showed the presence of lipid, amino acids and sugar fractions, revealing glycolipoprotein type of BSs. The BSs with glycolipopeptide nature is a rare type of example, and most of the studies reported the BSs as either glycolipid [19, 20] or glycoprotein [15, 16, 38]. There are three reports viz., Moldes et al. [18], Vecino et al. [39] and Morais et al. [40] which showed the molecular compositions of BSs as glycolipopeptide. In addition to TLC technique, the molecular composition of the BSs was also supported with FTIR spectroscopy. The FTIR is the most useful technique to investigate chemical bonds (functional groups) present in the unknown compound, and thus determines its chemical nature. The FTIR shows the stretch between 3400 to 2400 cm,^− 1^ confirming the presence of –OH groups [19] whereas the small peak at 2851 cm^− 1^ corresponding to the existence of C-H bond. Presence of proteins and sugar is comparable with previous researches [18, 19]. In addition, a report highlighting the structure of BS from *L. acidophilus* species, Tahmourespour et al. [41], showed proteinaceous type of structure of BS from *L. acidophilus* DSM 20079, where the peak at 1′653 cm ^− 1^ (AmI band: CAO stretching in proteins) and a peak at 1′480 cm ^− 1^ (AmII band: NOH bending in proteins). The BS showed comparatively higher amounts of protein and less polysaccharides in the structure. Determination of accurate molecular structure is extremely challenging [36] and is beyond the capacity of the current study. With the help of TLC and SDS-PAGE, we confirm the presence of protein in the BS obtained from *L. acidophilus*. The FTIR spectrum obtained in our study is comparable with FTIR spectrum of BSs obtained from *L. pentosus* [18, 39], suggesting glycolipoprotein nature of BSs. At commercial scale low yield of BS is major concern where high cost inputs are mandatory. Therefore, usage of renewable substrates like dairy industry, agro-industrial waste, coffee processing residues, animal fat waste, fruit processing industry, food processing industry has been recommended by several researchers [36]. *L. agilis* CCUG31450 produces 84 mg/L CABS in MRS medium. Whereas usage of cheese whey results increase in CABS up to 960 mg/L [16]. We are reporting comparatively lower yield (175 mg/L) from the strain used by us in the current study. Lactic acid bacteria (LAB) are mainly known for production of cell associated type BS. However, there are few reports suggesting production of cell free type BS. Both type of BS (cell free and cell associated or bound) can be produced simultaneously. It is important to note that the production is in very small quantities. The amount of CABS produced by LAB has been reported ranging between 20 to 100 mg/L [16].

Knowledge of various physical properties of BSs such as SFT, IFT, CMC, CA, EA and ES presents number of opportunities to explore them for undeniable applications proving the efficiency of BS. Unlike the most explored method (DuNouy Ring Method- to determine the efficiency of BS), we used a pendant drop technique to determine the profile of the drop of BS solution suspended in another liquid at equilibrium (due to the balance between gravity and surface force). A measure of a decrease in the IFT value between liquid and kerosene interphase by BS is an indication of the effectiveness of BS, which can be explored further to remediate oil spills in oceans and petroleum contaminations in soils. The CA determination between BS and surfaces is an important to determine the wettability. The CMC determination is important because it represent the concentration of BSs at which no further reduction in the SFT value of BSs solution occurs despite an increase in the concentration of BSs. Therefore, lower the CMC value, more efficient the BSs. In the present study, the CMC value of BS was quite close to the CMC of SDS, indicating an efficient SFT reducing ability of BS derived from *Lactobacillus*. Most of the literature on SFT values for BS produced by *Lactobacilli* species are in the range of 41 to 46 mN/m with varied CMC values [15, 16, 21]. The CA determination is important since it defines the wetting characteristics of BS; the latter is an important for determining the degree of adhesion or affinity of a liquid for a solid surface. The wetting properties of BS are important as it dictates the adhesion of pathogens to biomaterials implants in human body. The CA measurements for BS from *Lactobacillus* spp. have not been frequently reported in literature. The ability of BS to reduce the CA on hydrophobic surfaces has been successfully utilized to interfere the initial adhesion of uropathogenic bacteria and yeasts on silicone based surfaces [42]. This has been demonstrated effectively on PDMS based surfaces in our present studies. Similarly, BS derived from *Lactobacilli* showed some good emulsification abilities as well as stabilities which is in an agreement with Portilla-Rivera et al. [43] for emulsions of octane/water stabilized by BS derived from *L. pentosus*. The agar double diffusion tests performed to know the ionic characteristics of BS revealed an anionic nature of BS derived from *Lactobacillus.* The test is actually an indication of the passive diffusion of two compounds having same or opposite types of charges in a weakly-concentrated gel. Anionic nature of BS (in present study) has also been reported by Sharma et al. [20] for BS derived from *L. helveticus* strain MRTL91.

After studying the physical, molecular, properties of BS, we decided to investigate the antibacterial, antiadhesive and antibiofilm properties. The study of antiadhesive and antibiofilm activities of BS become essential due the reports of the failure of bio-implants due to colonization of biofilm forming organisms, and an increased resistance of such microorganisms (due to biofilm) to the treatment of antibiotics. The improved antiadhesive and antibiofilm activities of BS from *Lactobacillus* could be due the molecular and physiological characteristics of biofilm. Hydrophobicity and surface charge of a bacterial cell are important factors that govern the non-specific attachment of the bacteria to a surface. It is well known that most of microorganisms possess negative charges; therefore, the antiadhesion property shown by BS in present study could be due to an anionic nature of BS, which could have repelled the microbial adherence to the surfaces. The physical forces responsible for bacterial adhesion to surfaces include steric interactions, van der Waals forces and electrostatic (double layer) interaction. Also alteration/ fluctuation in the microenvironment make it difficult for bacterial cells to adapt easily. Gram negative bacteria tend to adhere to moderately hydrophobic surfaces owing to their hydrophobic LPS (lipopolysaccharide) layer [44]. In addition, the roughness of the surface aids in adherence of bacteria. Coating the PDMS surface with CABS reduces the surface roughness hindering bacterial adhesion to the surface. Another reason for such antiadhesive property of BS could be due the wetting properties of BS (small CA) which could have altered the hydrophobicity, and thus prevented the initial attachment of microorganisms to surface. The CA of distilled water was found to be reduced slightly on the PDMS surface which was coated with BS. This reduction might be due to the presence of protein and sugar moieties and their interaction with water on the BS coated surface. Also, the analysis of reduction in bacterial adhesion on BS coated surface might be due to the alteration of surface properties of PDMS surface, however do not allow adherence of organisms to the surfaces of medical implants which might have altered the bacterial adhesion at the molecular level. The antibiofilm activities could be explained due to the ability of BS to reduce the SFT between biofilm and substratum on which biofilm forms, thus, dispersing the biofilm from substratum. Secondly, antimicrobial property of BS could also be the reason for the antibiofilm activity of BS. Present research reports antibiofilm and antiadhesive properties of *Lactobacillus* derived BS are in agreement with Gudiña et al. [16, 17, 45]. BS produced by other *Lactobacillus* spp. like *L. jensenii* and *L. rhamnosus* do exhibit antimicrobial, antibiofilm as well as antiadhesive properties against multidrug resistant pathogens [46, 47]. Our observation is comparable with Dusane et al. [48] who revealed that BS from marine bacterium *S. marcescens* exhibits antimicrobial activity against *C. albicans*, *P. aeruginosa* and *B. pumilus*. Similar studies carried out by Velraeds et al. [49] showed inhibitory effect of BS produced by *Lactobacillus* spp. on *E. faecalis* biofilms on glass surfaces. We investigated antibiofilm effect of BS on PDMS based surfaces where biofilms *P. vulgaris, S. aureus* and *B. subtilis* were nearly inhibited. Previous studies demonstrated by Mireles et al. [50] indicated the inhibition of biofilm through surfactin on vinyl urethral catheters. Our study demonstrates good antiadhesion abilities as compared with antibacterial properties which is more noteworthy. Many researchers have recommended a concentration of CABS ranging between 25 and 50 mg/ml [36]. Recent studies [51] demonstrates that *Lactobacillus* spp. derived BS exhibiting antioxidant and antiproliferative properties against biofilm producers indicating the promiscuous applications in prevention of oral diseases. BS produced by *Lactobacillus* spp. have offered its applications in cosmetic formulations and medicine [52, 53]. Thus *Lactobacillus* is proving to be exceptionally promising candidate for the designing of newer BS based products. The proposed fact of our studies can be supported by Brzozowski, et al. [21] where BS is associated with an inhibition of adhesion of pathogens where the adhesion mechanisms is crucial than direct antimicrobial activity. Antiadhesive potential of *L. acidophilus* BS can be utilized against used against one of the nosocomial infectious pathogen like *S. marcescens*. Infections from various microorganisms at vaginal, urinary and gastrointestinal tracts infections can be protected with the help of BS derived *L. acidophilus* [22]. We have obtained protein based BS and such compounds are highly helpful in preventing the adhesion of pathogens. Our opinion is in agreement with Velraeds et al. 1998 [42] who have demonstrated the effective role of surlactin (protein rich CABS) derived from *L. acidophilus* RC14 as antiadhesive against various uropathogenic bacteria to silicone rubber surfaces. One of the pathogens namely *S. mutans* associated with dental caries and biofilm formations are effectively inhibited by the CABS produced by *L. acidophilus* [41]. Finally, we conclude that powerful BS is derived from *Lactobacillus* having huge potential as antiadhesive agent which can be used for various surfaces of biomedical devices.

## Conclusion

To the best of our knowledge, this is the first report extensively discussing the characterization of various physical properties of BS derived from *Lactobacillus* spp. and we are reporting the lowest surface tension value. We are also proposing a simple fermentation medium for BS production; where MRS medium was the routine practice. Our work demonstrated that BS from *L. acidophilus* exhibited antibiofilm and antiadhesive activities against biofilm producers on PDMS based medical implant surfaces.

## Abbreviations

BS: Biosurfactant
CA: Contact angle
CABS: Cell-associated-biosurfactant
CMC: Critical micelle concentration
FTIR: Flourier transform infrared spectroscopy
IFT: Interfacial tension
PBS: Phosphate buffer saline
PDMS: Polydimethylsiloxane
SDS-PAGE: Sodium Dodecyl Sulphate Polyacrylamine Gel Electrophoresis
SFT: Surface tension
TLC: Thin layered chromatography
